# Unraveling the Yellow Mystery: Severe Jaundice and Multisystem Collapse in a Returning Traveler

**DOI:** 10.7759/cureus.86217

**Published:** 2025-06-17

**Authors:** Bharadwaj Adithya Sateesh, Natalie Shammas, Ahmed Abdelfattah, Jarmil Alyssa Peters, Kristin Dishongh, Ashley Jenkins, John Mark P Pabona

**Affiliations:** 1 Internal Medicine, Catholic Health Initiatives (CHI) St. Vincent, Hot Springs, USA; 2 Pulmonary and Critical Care Medicine, Catholic Health Initiatives (CHI) St. Vincent, Hot Springs, USA; 3 Pathology, Catholic Health Initiatives (CHI) St. Vincent, Hot Springs, USA

**Keywords:** abdominal gas gangrene, acute edematous pancreatitis, cirrhosis, edematous pancreatitis, gas gangrene, jaundice, shock

## Abstract

We present the case of a 43-year-old male with recent travel history and possible toxic exposure abroad who was admitted with altered mental status following a seizure. His course was complicated by multiorgan dysfunction, including acute renal failure, rhabdomyolysis, acute pancreatitis, disseminated intravascular coagulation, and severe jaundice. Extensive infectious and autoimmune workup was unrevealing. Imaging revealed hepatosplenomegaly, hemorrhagic ascites, and progressive pancreatic inflammation. Liver biopsy demonstrated cirrhosis with cholestasis, ballooning degeneration, Mallory hyaline, and bridging fibrosis. Despite supportive care, the patient developed hemorrhagic pancreatitis with retroperitoneal gas, concerning for pancreatic gas gangrene. Surgical intervention was not feasible, and the patient ultimately succumbed to his illness. This case underscores the diagnostic complexity and rapidly fatal potential of overlapping hepatic and pancreatic pathologies in the setting of unclear toxic or environmental exposures.

## Introduction

Jaundice is a medical condition characterized by the development of yellow pigmentation of the sclera, mucous membranes, and skin due to hyperbilirubinemia [1]. Determining the underlying cause of hyperbilirubinemia can be challenging because of its wide range of etiologies, the most notable being intrahepatic and extrahepatic factors. In practice, differentiating the causes of jaundice requires a comprehensive history, physical examination, relevant laboratory tests, and diagnostic imaging. When incorporated altogether, these aid the physician in classifying jaundice into obstructive or nonobstructive subtypes [2]. Obstructive jaundice results from blocked bile ducts, preventing bile from flowing correctly from the liver to the gallbladder and digestive tract. An appropriate preoperative evaluation can significantly improve outcomes. However, in most cases, surgery for obstructive jaundice is associated with a higher incidence of complications and increased mortality rates [3]. Acute hepatic failure is an important cause of rapid-onset jaundice and altered mental status, which warrants urgent evaluation and supportive care [1]. Additionally, acute pancreatitis can contribute to extrahepatic biliary obstruction through inflammatory swelling or complications such as pseudocyst formation, highlighting the importance of appropriate cross-sectional imaging to evaluate for such obstructive etiologies [2]. We present a patient with jaundice who developed several unique complications, ultimately leading to a poor prognosis and mortality.

## Case presentation

**Figure 1:**
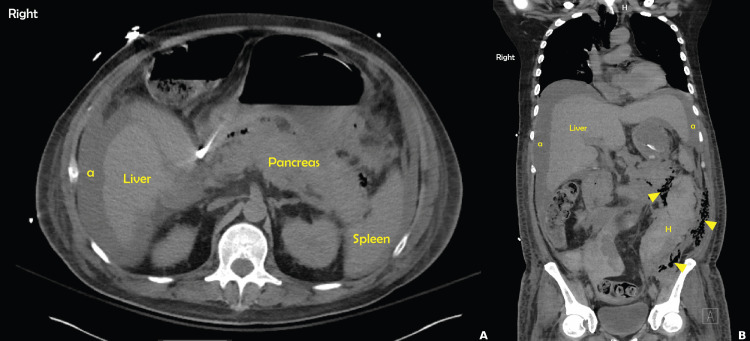
Abdomen and pelvis CT scan A: Axial section demonstrating diffuse interstitial pancreatic edema. There is also the presence of peri-hepatic ascites (a). B: Coronal section showing hemorrhage (H) in the retroperitoneum. There are also large amounts of gas surrounding the hemoperitoneum, which can be suspicious for gas gangrene (arrows). There is also a presence of abdominal ascites (a).

**Figure 2:**
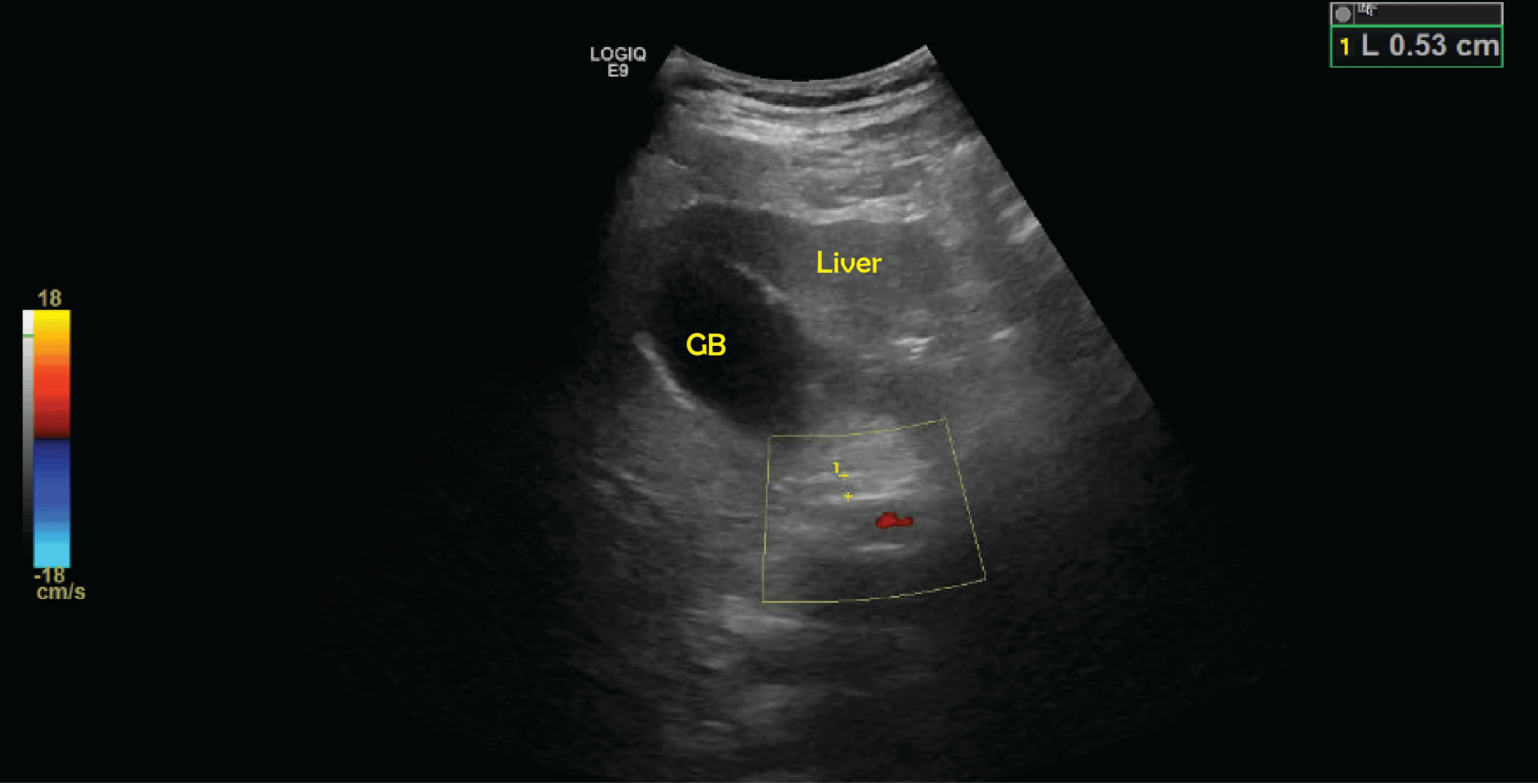
Ultrasound of the abdomen showing normal common bile duct (5.3 mm)

A 43-year-old male with a history of treated malaria, with military work as a private contractor in Southeast Asia, and possible exposure to unknown toxins abroad, presented with altered mental status following a seizure. He was subsequently intubated for airway protection with concerns of septic shock upon admission. He was found to be in acute renal failure, with rhabdomyolysis, acute pancreatitis, and disseminated intravascular coagulation (Tables 1-3). On physical examination, he was noted to have severe jaundice with scleral icterus. He was placed on broad-spectrum antibiotics (vancomycin and cefepime) as he underwent further testing. Computed tomography (CT) scan of the head was negative for any acute abnormalities. CT of abdomen and pelvis (A/P) did show hepatosplenomegaly, hemorrhagic ascites within the pelvis, and small hemorrhages within the bladder (Figures 1A, 1B). Lumbar puncture was conducted, which ruled out concern for meningitis. An extensive workup was completed, which included leptospirosis, tuberculosis, cryptococcal disease, tick-borne disease, hepatitis (A, B, and C), autoimmune hepatitis, ehrlichiosis, eastern equine encephalitis, Western equine encephalitis, California encephalitis, tuberculosis, malaria, dengue, HSV, John Cunningham virus, West Nile virus, CSF testing for multiple bacteria, viruses, and yeast, and a parasite panel, which were all negative. His labs continued to worsen (notably worsening leukocytosis, transaminitis, and hyperbilirubinemia) with consideration of possible initiation of continuous renal replacement therapy (CRRT); however, this was challenging as he remained on vasopressor support for treatment of shock, which was attributed to hypovolemic versus septic etiologies. His pancreatitis continued to worsen with no identifiable cause; repeat ultrasound of the abdomen did not reveal a concern for bile duct dilation (Figure 2). MRCP was suggestive of a possible hyperdense foci within the pancreas, possibly representing parenchymal hemorrhage, with a moderately distended gallbladder with sludge.

**Table 1:** Complete blood count values L: low; H: high; LL: low panic; HH: high panic

Day of admission	WBC (4.5-11.0 K/uL)	RBC (4.50-5.90 M/uL)	Hemoglobin (13.5-17.5 g/dL)	Hematocrit (41.0-53.0 %)	Platelets (150-400 K/uL)	MPV (7.4-10.4 fL)
Day 1	11.9 (H)	4.43 (L)	11.6 (L)	34 (L)	136 (L)	10.9 (H)
Day 2	5.2	3.60 (L)	8.8 (L)	25.9 (L)	61 (L)	11.6 (H)
Day 3	14.6 (H)	2.80 (L)	7.4 (L)	22.8 (L)	72 (L)	11.8 (H)
Day 4	14.2 (H)	2.47 (L)	7.9 (L)	23.9 (L)	60 (L)	12.7 (H)
Day 5	11.2 (H)	2.56 (L)	8.0 (L)	23.4 (L)	70 (L)	11.8 (H)
Day 6	8.8	2.44 (L)	7.7 (L)	22.3 (L)	59 (L)	13.2 (H)
Day 7	6.9	2.37 (L)	8.8 (L)	26 (L)	37 (LL)	10.4
Day 8	7.5	2.63 (L)	8.1 (L)	23.5 (L)	110 (L)	11.3 (H)
Day 9	7.8	2.83 (L)	8.6 (L)	25.0 (L)	101 (L)	12.0 (H)
Day 10	12.3 (H)	2.84 (L)	8.8 (L)	26.4 (L)	110 (L)	12.3 (H)
Day 11	15.7 (H)	2.80 (L)	8.7 (L)	26.7 (L)	141 (L)	12.7 (H)
Day 12	15.1 (H)	2.37 (L)	7.5 (L)	23.2 (L)	169	12.5 (H)
Day 13	20.8 (H)	2.68 (L)	8.3 (L)	25.7 (L)	235	12.0 (H)
Day 14	18.3 (H)	2.57 (L)	8.1 (L)	25.2 (L)	229	12.2 (H)
Day 15	15.2 (H)	2.27 (L)	7.0 (L)	22.4 (L)	257	11.3 (H)
Day 16	13.2 (H)	2.20 (L)	7.9 (L)	23.9 (L)	268	11.2 (H)
Day 17	12.1 (H)	2.60 (L)	8.1 (L)	24.2 (L)	242	11.1 (H)
Day 18	10.8	2.47 (L)	7.6 (L)	23.2 (L)	211	10.9 (H)
Day 19	9.5	2.29 (L)	7.1 (L)	21.4 (L)	193	11.2 (H)
Day 20	10.0	2.27 (L)	7.1 (L)	21.5 (L)	180	11.1 (H)
Day 21	13.5 (H)	2.50 (L)	8.0 (L)	23.3 (L)	268	11.6 (H)
Day 22	14.1 (H)	2.31 (L)	7.5 (L)	22.1 (L)	394	10.5 (H)
Day 23	17.7 (H)	3.00 (L)	9.3 (L)	27.8 (L)	488 (H)	10.5 (H)
Day 24	23.2 (H)	2.78 (L)	8.7 (L)	26.0 (L)	525 (H)	9.9
Day 25	18.0 (H)	2.76 (L)	8.7 (L)	25.4 (L)	505 (H)	9.5
Day 26	22.0 (H)	2.83 (L)	8.8 (L)	25.8 (L)	490 (H)	9.3
Day 27	16.7 (H)	2.30 (L)	5.9 (LL)	18.1 (LL)	308	9.8
Day 28	30.4 (HH)	2.11 (L)	7.6 (L)	23.0 (L)	359	10.2
Day 29	24.0 (H)	2.13 (L)	7.3 (L)	21.5 (L)	309	9.9

**Table 2:** Comprehensive metabolic panel values H: high; HH: high panic; L: low; LL: low panic

Day of admission	Sodium (136-145 mmol/L)	Potassium (3.5-5.1 mmol/L)	Creatinine (0.72-1.25 mg/dL)	Chloride (98-107 mmol/L)	CO2 (22-29 mmol/L)	Calcium (8.4-10.2 mg/dL)	BUN (7-26 mg/dL)	Glucose (74-99 mg/dL)	GFR (≥60 mL/min/1.73m²)	Anion gap (8-16 mmol/L)	Osmolality (275-295 mOsm/kg)	Total protein (6.4-8.3 g/dL)	Albumin (3.5-5.0 g/dL)	Bilirubin total (0.2-1.2 mg/dL)	Direct bilirubin (0.1-0.5 mg/dL)	ALP (40-150 U/L)	AST (5-34 U/L)	ALT (<55 U/L)
Day 1	141	4.4	3.00 (H)	104	12 (LL)	10.5 (H)	25 (H)	246 (H)	23 (L)	40 (H)	298 (H)	9.6 (H)	4.5	8.8 (H)	-	153 (H)	132 (H)	75 (H)
Day 2	141	2.8 (LL)	4.13 (H)	99	13 (LL)	6.2 (L)	25	325 (H)	17 (L)	29 (H)	293	6.3 (L)	3.0 (L)	7.9 (H)	-	109	142 (H)	53
Day 3	138	4.1	3.91 (H)	94 (L)	15 (LL)	5.0 (LL)	31 (H)	293 (H)	19 (L)	29 (H)	293	5.0 (L)	2.3 (L)	6.6 (H)	-	62	144 (H)	55 (H)
Day 4	138	4.2	4.28 (H)	93 (L)	14 (LL)	5.0 (LL)	34 (H)	279 (H)	17 (L)	31 (H)	296 (H)	5.5 (L)	2.8 (L)	6.7 (H)	-	52	145 (H)	49
Day 5	138	3.9	3.07 (H)	91 (L)	28	4.8 (LL)	40 (H)	150 (H)	25 (L)	19 (H)	288	5.6 (L)	2.5 (L)	7.6 (H)	-	69	122 (H)	39
Day 6	141	3.6	3.00 (H)	92 (L)	30 (H)	6.0 (LL)	46 (H)	131 (H)	26 (L)	19 (H)	295	6.0 (L)	2.9 (L)	10.0 (H)	-	163 (H)	142 (H)	53
Day 7	147 (H)	2.6 (LL)	2.54 (H)	98	30 (H)	6.0 (LL)	82 (H)	145 (H)	31 (L)	19 (H)	320 (H)	6.0 (L)	2.9 (L)	11.3 (H)	-	302 (H)	144 (H)	55 (H)
Day 8	150 (H)	2.9 (LL)	1.69 (H)	99	33 (H)	6.0 (LL)	93 (H)	208 (H)	51 (L)	18 (H)	333 (H)	6.2 (L)	2.6 (L)	6.9 (H)	-	63	151 (H)	51
Day 9	154 (H)	3.3 (L)	1.26 (H)	101	36 (H)	6.2 (L)	94 (H)	208 (H)	>60	17 (H)	341 (H)	6.2 (L)	2.4 (L)	16.5 (H)	-	391 (H)	81 (H)	81 (H)
Day 10	151 (H)	3.5	0.89	106	31 (H)	6.3 (L)	87 (H)	145 (H)	>60	14	329 (H)	5.6 (L)	2.0 (L)	21.3 (HH)	-	314 (H)	41 (H)	62 (H)
Day 11	151 (H)	4.1	0.80	115 (H)	25	7.7 (L)	54 (H)	136 (H)	>60	11	317 (H)	5.4 (L)	2.0 (L)	23.6 (HH)	-	263 (H)	53 (H)	53
Day 12	146 (H)	5.0	0.91	115 (H)	22	8.0 (L)	47 (H)	197 (H)	>60	9	308 (H)	5.7 (L)	1.8 (L)	23.2 (HH)	-	212 (H)	45 (H)	46
Day 13	148 (H)	4.0	0.99	114 (H)	20 (L)	8.3 (L)	44 (H)	93	>60	14	305 (H)	5.8 (L)	2.2 (L)	22.5 (HH)	-	217 (H)	44 (H)	47
Day 14	149 (H)	4.0	0.89	116 (H)	21 (L)	8.3 (L)	42 (H)	82	>60	12	306 (H)	5.9 (L)	2.2 (L)	20.2 (HH)	-	240 (H)	52 (H)	51
Day 15	149 (H)	3.8	0.78	119 (H)	20 (L)	8.1 (L)	37 (H)	116 (H)	>60	10	306 (H)	5.8 (L)	2.3 (L)	25.1 (HH)	13.1	288 (H)	51 (H)	48
Day 16	153 (H)	3.7	0.70 (L)	122 (HH)	19 (L)	8.1 (L)	30 (H)	138 (H)	>60	12	312 (H)	5.6 (L)	2.3 (L)	26.0 (HH)	11.2	329 (H)	52 (H)	52
Day 17	148 (H)	3.1 (L)	0.70 (L)	118 (H)	19 (L)	7.7 (L)	28 (H)	146 (H)	>60	11	302 (H)	5.4 (L)	2.3 (L)	26.1 (HH)	10.5	357 (H)	61 (H)	50
Day 18	144	3.4 (L)	0.68 (L)	115 (H)	23	8.0 (L)	27 (H)	124 (H)	>60	6 (L)	293	5.7 (L)	2.3 (L)	25.0 (HH)	10.4	366 (H)	57 (H)	48
Day 19	138	3.9	0.63 (L)	109 (H)	21 (L)	8.2 (L)	24	127 (H)	>60	8	281	5.8 (L)	2.2 (L)	24.1 (HH)	11.7	371 (H)	56 (H)	44
Day 20	137	3.8	0.66 (L)	106	21 (L)	8.1 (L)	25	125 (H)	>60	10	280	5.9 (L)	2.3 (L)	25.4 (HH)	13.8	422 (H)	63 (H)	51
Day 21	133 (L)	3.5	0.64 (L)	103	20 (L)	8.0 (L)	25	126 (H)	>60	10	272 (L)	6.0 (L)	2.3 (L)	30.4 (HH)	>15	415 (H)	148 (H)	108 (H)
Day 22	131 (L)	4.4	0.61 (L)	100	19 (L)	8.0 (L)	27 (H)	250 (H)	>60	12	276	6.2 (L)	2.3 (L)	33.1 (HH)	>15	588 (H)	178 (H)	172 (H)
Day 23	134 (L)	3.7	0.72	103	22	8.4	44 (H)	161 (H)	>60	9	283	6.2 (L)	2.8 (L)	23.7 (HH)	>15	618 (H)	113 (H)	151 (H)
Day 24	137	3.8	0.69 (L)	108 (H)	21 (L)	8.5	31 (H)	113 (H)	>60	8	281	5.5 (L)	2.2 (L)	15.0 (H)	>15	609 (H)	129 (H)	174 (H)
Day 25	135 (L)	4.3	0.27 (L)	106	19 (L)	8.6	7	186 (H)	>60	10	273 (L)	5.9 (L)	2.6 (L)	13.6 (H)	>15	525 (H)	111 (H)	184 (H)
Day 26	134 (L)	4.1	0.62 (L)	107	20 (L)	8.8	24	163 (H)	>60	7 (L)	276	5.6 (L)	2.4 (L)	13.3 (H)	>15	516 (H)	107 (H)	200 (H)
Day 27	137	3.7	0.60 (L)	106	20 (L)	8.4	28 (H)	167 (H)	>60	11	283	5.6 (L)	2.3 (L)	15.3 (H)	>15	572 (H)	174 (H)	166 (H)
Day 28	132 (L)	4.0	0.60 (L)	104	18 (L)	8.4	26	163 (H)	>60	10	273 (L)	5.3 (L)	2.2 (L)	18.5 (HH)	>15	657 (H)	204 (H)	309 (H)
Day 29	131 (L)	3.9	0.56 (L)	103	18 (L)	8.3 (L)	28 (H)	196 (H)	>60	10	274 (L)	5.3 (L)	2.2 (L)	24.7 (HH)	>15	575 (H)	170 (H)	318 (H)
Day 30	130 (L)	3.5	0.63 (L)	103	18 (L)	8.6	22	150 (H)	>60	9	267 (L)	5.4 (L)	2.2 (L)	25.1 (HH)	> 15	539 (H)	1112 (H)	993 (H)
Day 31	131 (L)	4.8	0.91	104	8 (LL)	9.1	38 (H)	200 (H)	>60	19 (H)	277	4.8 (L)	2.3 (L)	30.4 (HH)	> 15	669 (H)	191 (H)	316 (H)
Day 32	132 (L)	5.2 (H)	1.47 (H)	98	7 (LL)	8.9	51 (H)	218 (H)	60	27 (H)	285	4.7 (L)	2.3 (L)	33.1 (HH)	> 15	669 (H)	191 (H)	316 (H)

**Table 3:** Coagulation panel values L: low; H: high; LL: low panic; HH: high panic

Day of admission	Prothrombin time (9.5-12.1 sec)	INR (0.9-1.1)	PTT (24.0-32.0 sec)	Fibrinogen (200-400 mg/dL)	D-dimer (0.00-0.50 ug/mL FEU)
Day 1	12.3 (H)	1.2 (H)	35.5 (H)	-	-
Day 2	>90.0 (HH)	-	29.1	110 (L)	4.14 (H)
Day 3	15.9 (H)	1.5 (H)	45.6 (H)	145 (L)	14.23 (H)

**Figure 3:**
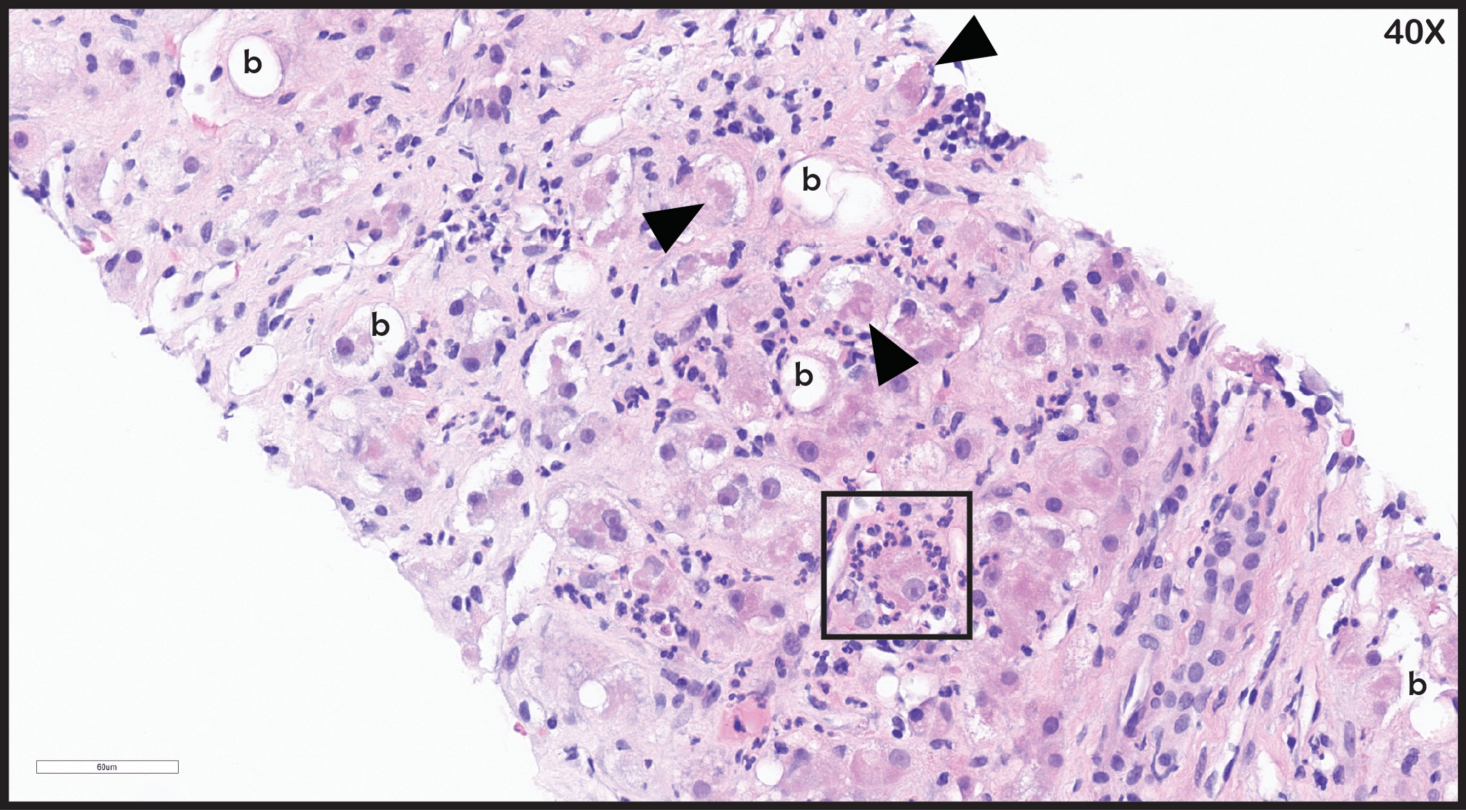
Liver biopsy H&E staining Hepatic steatosis with ballooning degeneration (b), Mallory hyaline (black arrowheads), and neutrophilic satellitosis (box).

**Figure 4:**
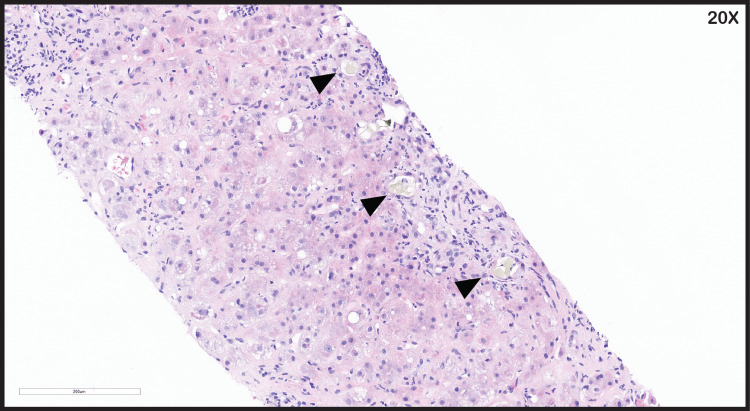
Liver parenchyma replaced with areas of canalicular cholestasis (black arrowheads)

**Figure 5:**
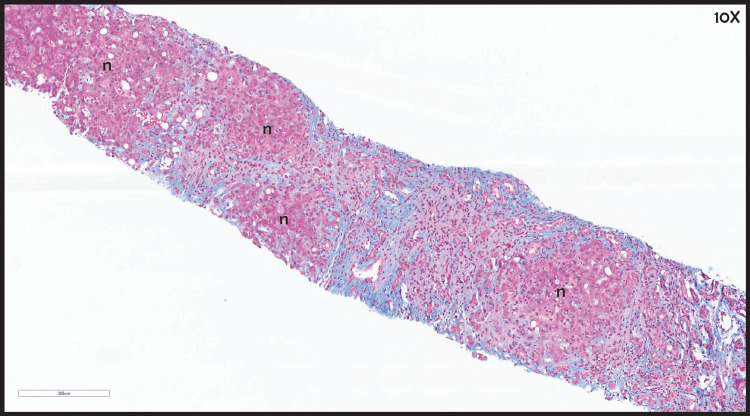
Liver biopsy with trichrome staining Cirrhotic liver demonstrating bridging fibrosis between regenerative nodules (n).

**Figure 6:**
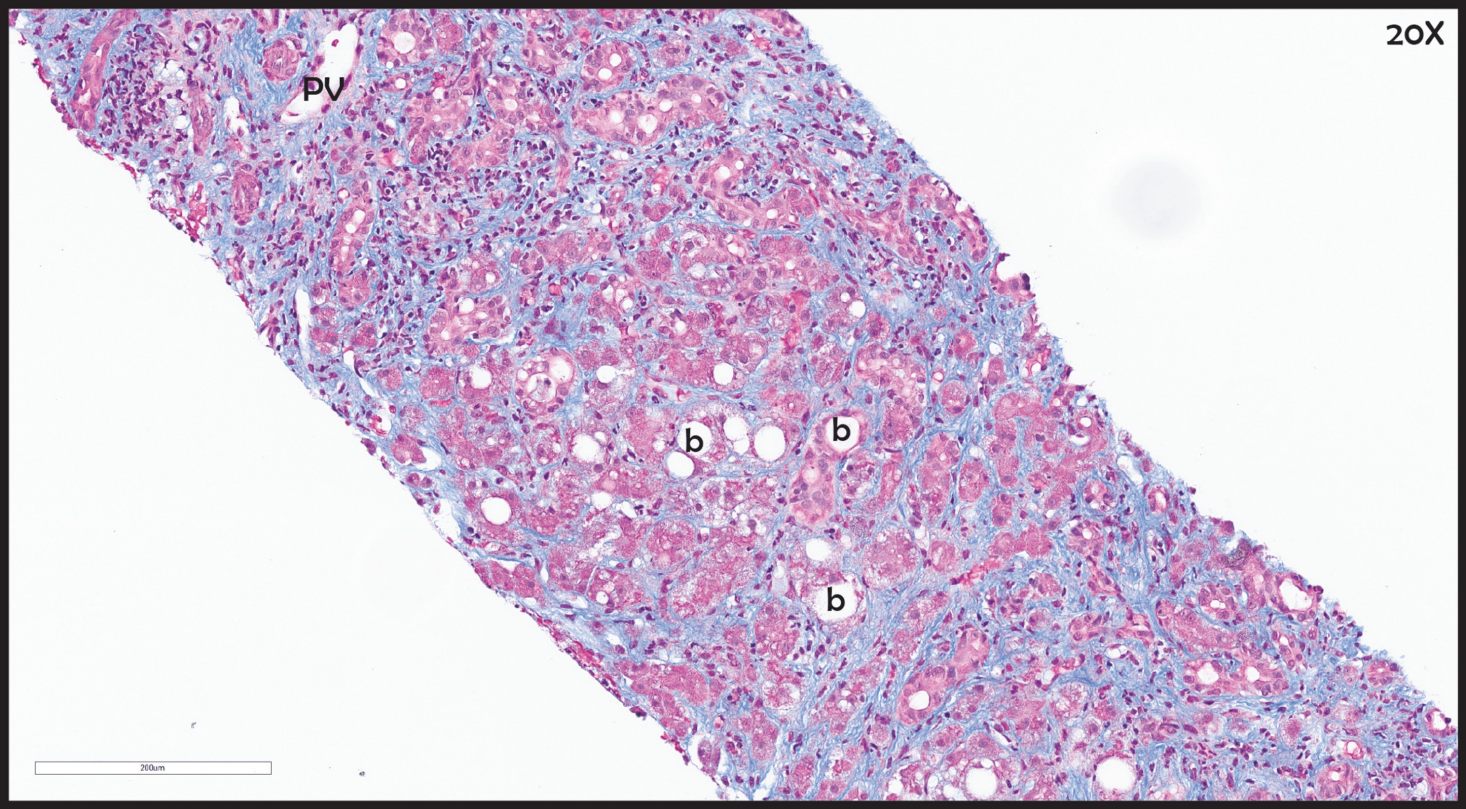
Diffuse fibrosis within liver parenchyma with areas of ballooning degeneration (b) Portal tracts and fibrous bands contain a mixed inflammatory infiltrate. PV, portal vein

A liver biopsy by interventional radiology was completed, showing liver cirrhosis with cholestasis. Hematoxylin and eosin staining revealed ballooning degeneration of hepatocytes, with Mallory hyaline and neutrophilic steatosis (Figure 3). There were also areas of cholestasis in the liver parenchyma (Figure 4). Upon trichrome staining, the liver demonstrated bridging fibrosis (Figure 5), and on 20× magnification, the liver parenchyma showed diffuse fibrosis with ballooning degeneration (Figure 6).

It was discovered that the patient did in fact have a history of alcohol use in the past; however, the extent of use could not be determined. Within the next several days, he became hypotensive. A repeat CT abdomen and pelvis showed acute interstitial edematous pancreatitis complicated by hemorrhage in the retroperitoneum and hemoperitoneum with large amounts of gas suspicious for gas gangrene. Surgery was consulted; however, he was deemed not a surgical candidate. Due to his poor prognosis, the family decided to proceed with withdrawal of care, and he subsequently passed.

## Discussion

Acute pancreatitis is an inflammatory condition of the pancreas that can range from mild to severe, with severe cases often leading to multiorgan dysfunction syndrome (MODS) [4]. Systemic inflammatory response syndrome (SIRS) triggered by acute pancreatitis can result in widespread inflammation, leading to organ failure [5]. A study by Mofidi et al. demonstrated a strong association between early systemic inflammatory response, severity of multiorgan dysfunction, and mortality in acute pancreatitis [4]. Respiratory failure is one of the most common organ dysfunctions observed in severe acute pancreatitis, occurring in approximately 23% of cases and significantly increasing mortality risk [5]. Renal failure is another critical complication, often resulting from hypovolemia, systemic inflammation, and direct effects of pancreatic enzymes [6]. Both respiratory and renal failures significantly impact patient outcomes, with cardiovascular failure leading to the worst prognosis [6]. In this patient, the intense inflammatory cascade and cytokine-mediated microcirculatory impairment likely contributed to hepatic decompensation by worsening hepatocellular injury, cholestasis, and synthetic dysfunction [4-6].

A particularly rare and fatal complication of acute pancreatitis is gas gangrene of the pancreas, often caused by *Clostridium perfringens* infection. This condition is characterized by the presence of gas within the pancreatic parenchyma and surrounding retroperitoneal tissues, leading to rapid tissue necrosis and systemic toxicity [7]. A case reported by Stockinger and Corsetti described a patient with acute pancreatitis complicated by pancreatic gas gangrene, resulting in pneumoperitoneum without visceral perforation [7]. A case reported by Deręgowska-Cylke et al. highlighted the development of emphysematous pancreatitis associated with retroperitoneal gas gangrene, emphasizing the severity and rapid progression of this infection [8].

Gas gangrene is commonly associated with *Clostridium* species, which are known for their ability to produce gas and extensive tissue necrosis. A retrospective epidemiological study by Leal et al. demonstrated that *Clostridium* bacteremia, particularly involving C. perfringens, carries a high mortality rate due to its rapid progression and limited treatment options [9]. While other possible causes of gas in the retroperitoneum, such as emphysematous pancreatitis, enteric perforation, or fistulous connections, should be considered, in this case, no visceral perforation was identified on imaging, and the rapid clinical deterioration supported a diagnosis of gas gangrene [7,8]. Blood cultures in our patient did not yield *Clostridium* or other pathogens, and ascitic fluid was not tapped due to hemodynamic instability and coagulopathy. Therefore, based on the extensive retroperitoneal gas and hemorrhagic necrosis on imaging, gas gangrene was presumed to be secondary to a spontaneous *Clostridium* infection.

The management of severe acute pancreatitis complicated by infections like gas gangrene requires prompt recognition and aggressive intervention. Early fluid resuscitation, broad-spectrum antibiotics, and supportive care in an intensive care setting are critical [10]. However, the presence of gas-forming infections often necessitates surgical intervention. 

Liver cirrhosis affected approximately 2.2 million adults in the United States from 2010 to 2021, with a 10% increase in mortality rate [11]. Most common causes are alcohol abuse, nonalcoholic fatty liver disease, and hepatitis C, none of which matched the history provided by our patient, except prior consumption of alcohol. Usually, patients with cirrhosis present with encephalopathy and ascites and are often diagnosed with cirrhosis through these presenting complications [11]. Further complications include spontaneous bacterial peritonitis, hepatorenal syndrome (median survival of two weeks), and hepatocellular carcinoma. Although our patient was found to have cirrhosis likely secondary to alcohol use, there were no findings of hepatocellular carcinoma on biopsy. The injury to the liver could likely have been multifactorial in the above patient, given his line of work, leading to potential toxins abroad, alcohol use, and subsequent systemic inflammatory response, further emphasizing the diagnostic complexity in the patient’s case. Potential hepatotoxin exposure in Southeast Asia includes traditional herbal remedies, aflatoxin-contaminated foods, and environmental or occupational chemicals, such as organic solvents (e.g., carbon tetrachloride and trichloroethylene), pesticides, and heavy metals like arsenic [12]. While liver biopsy can provide valuable histopathological insights, its diagnostic specificity in differentiating alcoholic hepatitis from toxin-induced liver injury remains limited. Both conditions can share overlapping features such as ballooning degeneration, Mallory-Denk bodies, neutrophilic infiltration, and varying degrees of fibrosis and cholestasis [13]. In this case, the diagnostic sensitivity of abdominal ultrasound may have been limited by factors such as operator dependency, patient body habitus, and suboptimal acoustic windows, which can reduce its ability to detect subtle biliary obstruction or small mass lesions. This is why repeat imaging with CT was performed to provide improved anatomic detail. Consequently, distinguishing between these etiologies purely on histological grounds can be challenging without a clear clinical context and corroborating history of alcohol consumption or toxin exposure, making it complex for the clinician. Management is largely targeted at the above-mentioned complications of cirrhosis with nonselective beta-blockers, spironolactone, lactulose, and paracentesis. 

In the case presented, the patient’s poor surgical candidacy due to multiorgan failure limited treatment options, leading to a fatal outcome. The importance of early detection and management of complications in severe acute pancreatitis with associated multiorgan dysfunction is vital.

## Conclusions

This case report highlights the complexities and challenges of diagnosing and managing severe obstructive jaundice complicated by multiple organ system failures and a rare but fatal gas gangrene of the pancreas. The patient’s history of travel, possible toxin exposure, and underlying liver cirrhosis further complicated the clinical picture. Despite extensive testing and supportive care, the patient’s condition deteriorated, leading to a poor prognosis. This case underscores the critical importance of early detection, aggressive intervention, and multidisciplinary management in patients with severe acute pancreatitis and associated multiorgan dysfunction to improve outcomes. This case also highlights the diagnostic uncertainty clinicians face in patients with overlapping hepatic and pancreatic findings, particularly when toxin exposure is unclear and lab data are nonspecific. Furthermore, it emphasizes the need for a thorough investigation of jaundice etiologies, particularly in patients with complex medical histories and risk factors.
